# A multi-level study on whether ethical climate influences the affective well-being of millennial employees

**DOI:** 10.3389/fpsyg.2022.1028082

**Published:** 2022-10-21

**Authors:** Wei Su, Juhee Hahn

**Affiliations:** ^1^The Graduate School, Chung-Ang University, Seoul, South Korea; ^2^Department of Business Management, Chung-Ang University, Seoul, South Korea

**Keywords:** affective well-being, ethical climate, millennials, moral identity, organizational citizenship behavior

## Abstract

Millennial employees are increasingly paying more attention to well-being in the workplace and it has become an important issue for managers. Given that millennial employees are more sensitive to ethical issues, this study began by analyzing an ethical element in the organization—the ethical climate—and explored whether millennial employees have higher affective well-being in organizations with a good ethical climate. We verified our hypotheses based on 288 valid questionnaires collected from 40 teams. The results showed that: (1) ethical climate was a positive predictor of millennial employees’ organizational citizenship behavior (OCB) and affective well-being, (2) employees’ OCB partially mediated the relationship between ethical climate and affective well-being, and (3) an employee’s moral identity effectively moderated the relationship between ethical climate and affective well-being, although it did not play a significant moderating role between ethical climate and OCB. These findings provide empirical support for applying situational strength and social information processing theories and emphasize the importance of cultivating an ethical climate in organizations.

## Introduction

People born between 1980 and 2000 are known as millennials, and it is estimated that by 2025, millennials will comprise 75% of the global workforce (Brant and Castro, 2019). Several studies have raised the importance of the subjective experiences of millennial employees in the workplace, suggesting that as a cohort they tend to place more value on their emotional experiences when evaluating their overall satisfaction, performance levels, and decision to remain with an organization (García et al., 2019; He et al., 2019). Millennial employees who experience high levels of affective well-being at work are more likely to be more creative, resilient, and socially competent (Badri et al., 2022). More importantly, they are more likely to remain committed to their work and organization (Yuniasanti et al., 2019). Therefore, improving the affective well-being of millennial employees has become a key challenge for managers.

Millennials have grown up in an age when seemingly no behavior goes unnoticed or unreported. There is now a 24-h news cycle, increased government oversight, and a significant increase in reporting of large-scale ethical scandals (e.g., the 2008 milk scandal), while social media has become all-pervading (VanMeter et al., 2013). The Generational Differences in Workplace Ethics survey, which was conducted by the Ethics Resource Center, shows that millennials notice misconduct in the workplace more than previous generations. In addition, 67% would choose to report their observed misconduct, compared with 39% of older workers. According to Ernst and Young’s (2017) Asia-Pacific Fraud Survey Report, more than 80% of millennials—the largest proportion compared with other generations—expressed reluctance to continue working for organizations that were involved in unethical practices, such as fraud, bribery, and corruption. Previous research has demonstrated that millennials also value clear and ethical rules and expectations (Curtin et al., 2011). Having a clear value statement, which is known to employees and evidenced in the workplace, is key to workplace satisfaction among millennials (Bowen, 2010). As the ethical climate in the workplace is an ethical guideline shared within an organization and defines ways of dealing with ethical issues, we believe that the ethical climate of a workplace is a concern of millennial employees. Emotions can arise in response to specific environmental events, according to the cognitive theory of emotions which states that cognition triggers emotions (Lazarus, 1991). However, the difference between negative and positive emotions is one of the personal assessments regarding the particular event’s impact on the individual’s goals and values (Bagozzi et al., 2003). Thus, events that a person evaluates as consistent with their goals or values can trigger positive emotions (e.g., happiness, contentment, and pride), while events at odds with one’s goals or values can trigger negative emotions (e.g., anger, sadness, and shame; Wijewardena et al., 2014). Therefore, since millennials are perceived to be sensitive to ethical issues, we also believe that millennials will experience more positive emotions in an organization with a good ethical climate.

An ethical climate can be defined as a moral consensus shared by employees, while affective well-being can be thought of as an employee’s psychological and emotional experience. Since both of these are intangible spiritual experiences, we need to develop a tangible behavior that can connect the two. In addition, as the ethical climate is an essential aspect of organizational culture, it can directly influence how people behave (Teresi et al., 2019). Organizational citizenship behavior (OCB) is a voluntary and entirely selfless activity that employees undertake outside of their job responsibilities, significantly impacting the organization. OCB is also the most common and accessible extra-role pro-organizational behavior visible in the workplace. Previous research has shown that pro-social behavior can effectively boost mood (Guo et al., 2018), although few studies have investigated whether OCBs are directly related to the health and well-being of employees (Baranik and Eby, 2016). This study, therefore, adopts a resource-rich view and focuses on the potentially positive impacts of OCB on well-being. The extant literature on OCB has not widely discussed this view (Lam et al., 2016). Many previous studies have examined the relationship between ethical climate and OCB (Dinc and Aydemir, 2014; Aloustani et al., 2020), but few studies have combined OCB and ethical climate with employees’ affective well-being. Therefore, this study uses OCB as a mediator variable to further explore the relationship between OCB and the affective well-being of millennial employees.

Recent research has also shown that the impact of an ethical climate on employees will vary depending on their personalities (Al Halbusi et al., 2020). When the values of the individual and the organization coincide, employees will show more positive attitudes (Wu et al., 2020). Moral sensitivity is affected differently by situational and individual factors, with individual characteristics (moral identity) having a significant impact on moral sensitivity (Sparks, 2015). Therefore, this study will contribute to this growing body of literature by testing whether employees’ moral identities will moderate the impact of the ethical climate on OCB and affective well-being.

The overall objective of this study is to explore the relationship between ethical climate and millennial employees’ affective well-being and to establish a framework for confirming OCB’s mediating role and moral identity’s moderating role. This research extends the available literature on ethical climate as an organizational variable since relatively few studies have analyzed the effect of ethical climate on psychological state compared with other outcome variables (Newman et al., 2017). Previous research has focused more on the impact of ethical leadership on employees’ affective well-being (Ahmad, 2019; Kaffashpoor and Sadeghian, 2020). However, ethics is a group-level phenomenon that can shape an organization’s internal relations and employee attitudes through ethical climates (Naber and Moffett, 2017). This study is a response to Newman et al. (2017) call to use situational strength theory (SST) to broaden our understanding of ethical climate. We have conducted an in-depth analysis of the relationship between ethical climate and affective well-being, and the mediating role of OCB. Previous studies have analyzed OCB from a resource consumption perspective and have shown that OCB consumes resources and fosters negative emotions. Positive psychology posits that positive and negative emotions can co-exist, and cannot be viewed as a dichotomy. From a resource-rich perspective, this study predicts that millennials can realize the value of helping others and gain a greater sense of work meaning through OCB, thereby increasing their affective well-being. This will also expand the previous hypothesis of the relationship between OCB and well-being. Previous studies have examined the relationship between ethical climate and OCB (Dinc and Aydemir, 2014; Aloustani et al., 2020) and the relationship between OCB and well-being (Baranik and Eby, 2016; Kaur and Kang, 2019). However, to the best of our knowledge, no study has conducted a multi-level model analysis that combines the three factors and the cross-level moderation of moral identity. We believe that this study is the first to verify the moderating role of moral identity as a personal characteristic in the relationship between ethical climate and affective well-being, which provides empirical evidence for the person-organization (P-O) fit theory. These findings of this study will provide some practical implications for managers who hope to boost the well-being of their millennial employees.

## Literature review and hypotheses development

### The ethical climate and employees’ affective well-being

Qualls and Puto (1989) proposed that the operationalization of the ethical climate measures individuals’ perceptions of the procedures, practices, values, and norms that govern ethical decision-making within an organization. We will therefore use Mayer et al. (2010, p. 7) definition in this study, which refers to ethical climate as “the holistic impression that individuals have regarding ethical policies, practices, and procedures within a unit or organization.” An organization’s ethical climate comprises the common normative beliefs and values of its employees regarding ethical issues. It can also be thought of as a moral code—behavioral principles that drive community and organizational perceptions of right and wrong. This research assesses the presence and implementation of ethical codes and policies and management actions related to ethics within an organization through seven-item scales.

Situational strength is defined as “implicit or explicit cues provided by external entities regarding the desirability of potential actions” (Meyer et al., 2010, p. 122). Strong situations have clear cues and clear behavioral expectations for reducing situational ambiguity (Newman et al., 2017). Situational intensity theory states that a strong ethical climate involves communicating clear and consistent information regarding the scope of ethical behavior that an organization considers acceptable and enforcing it by providing positive consequences for adherence and negative consequences for violations. An employee can feel uncertain about their moral obligations if there are no clear and conventional moral standards within their organization (i.e., the ethical climate)—an uncertainty that can result in vague and ambiguous ethical expectations. Role ambiguity is the most widely recognized source of psychological strain (De Clercq et al., 2019) and often occurs when employees are uncertain about their job expectations and responsibilities (Low et al., 2001). Researchers define the ethical climate of a workplace as the common opinion of “what is ethically acceptable behavior” and how ethical problems should be managed and controlled in the workplace. An ethical climate determines decision-making, moral criteria for understanding, and employees’ behavior in response to ethical issues, and helps employees to solve their moral problems by providing definitive guidance on what they should do (Naz et al., 2019). Researchers believe that when employees know what rules and procedures guide their actions, they perceive an absence of ambiguity within themselves (Martin and Cullen, 2006). Therefore, according to SST, an explicit ethical climate will reduce employees’ ambiguity, thereby reducing psychological pressures and improving affective well-being. In addition, ethical climates based on principle-centered criteria would facilitate decision-making based on organizational codes and regulations, reducing uncertainty and favoritism.

According to the stated moral principles, people reward ethical behaviors and punish unethical ones to ensure fairness (Agrawal, 2017). Organizational justice is one of the factors affecting employee well-being (Heffernan and Dundon, 2016). The ethical climate in a particular workplace forms a group experience where employees feel free to discuss ethical issues with their peers and management, and always feel supported when facing a moral dilemma (Snell et al., 2010). This type of support for ethical behavior can increase job satisfaction (Yang, 2014). By contrast, if employees perceive the ethical climate as weak, they will perceive a lack of support from their organization in meeting normative expectations and discussing ethical issues. Organizations with an unethical climate may pressure employees to engage in unethical behaviors, resulting in distress and dissatisfaction whenever an ethical conflict arises (Huhtala et al., 2016). Zhou et al. (2018) research found that a strong and unambiguous ethical climate enhances cognitive and emotional bonds between employees and the organization. If the organization upholds ethical values, norms, and beliefs, the ethical climate can promote positive interaction among employees and increase job satisfaction (a measure of job-related affective well-being; Domino et al., 2015; Hsieh and Wang, 2016). Therefore, we propose the following hypothesis.

*H1*: The ethical climate is positively related to employees’ affective well-being.

### Ethical climate and employees’ OCB

As mentioned earlier, OCBs are voluntary and altruistic activities performed by employees outside of their job responsibilities for which they may not get paid or rewarded (Podsakoff et al., 2000). OCB includes “contributions to the maintenance and enhancement of the social and psychological context that supports task performance” (Organ, 1997). There are five aspects to OCB: civic virtue, which Fassina et al. (2008) briefly summarize as follows: (1) altruism, in which the individual selflessly helps other employees in an organization, such as helping new employees adapt to the workplace; (2) courtesy, preventing colleagues from encountering problems and troubles, and informing them of precautions in advance; (3) conscientiousness, wherein employees show positive behaviors outside of company regulations, such as proactively protecting organizational resources; (4) civic virtue, wherein employees show a positive attitude and sense of responsibility toward company activities, such as actively participating in organizational meetings; and (5) sportsmanship, where employees do not think or act negatively in the workplace. (e.g., they will not complain about any minor inconveniences).

Social information processing theory shows how individuals utilize key cues and information from their surroundings to understand how to act appropriately in a particular environment (Salancik and Pfeffer, 1978); it is the core theory that analyzes the effect of ethical climate on employees’ OCB. When applying this theory to the workplace, employees would collect important information and cues from their surroundings and make suitable decisions or take action accordingly. Employees can observe, experience, and interpret more ethical behaviors when immersed in a conducive ethical climate, allowing them to behave in a manner that caters to their organization’s ethical values (Teng et al., 2020). Furthermore, teams with a highly ethical climate may reinforce external formal systems by rewarding ethical behavior or punishing unethical behavior. Thus, these tangible external rewards reinforce employees’ motivations for pro-social behavior (Bai et al., 2019). Similarly, other team members may be rewarded for ethical behavior or punished for unethical behavior, which will allow them to learn and behave in accordance with their team’s ethical climate (e.g., participating in pro-social behaviors such as OCB) (Aloustani et al., 2020). Therefore, when employees believe that their team’s climate is ethical, their ethical decision-making and behaviors are more likely to be affected. Employees who work in a highly ethical climate have greater ethical awareness, pay more attention to ethical issues, and engage in more OCB (Newman et al., 2017). Previous research has shown that a highly ethical climate positively impacts OCB (Çavuş and Develi, 2017; Lee and Ha-Brookshire, 2018) and positively mediates the relationship between leadership and OCB (Sendjaya et al., 2019; Fatima and Siddiqui, 2020). Based on the above reasoning, this study proposes that group members who observe similar social influences and clues in a common ethical climate will be more likely to participate in OCB.

*H2*: Ethical climate is positively related to employees’ OCB.

### Mediation of OCB on the relationship between ethical climate and employees’ affective well-being

This study posits that OCB can increase the meaningfulness of work and enrich personal resources, thereby enhancing individual affective well-being. When employees are more involved in OCB, they feel more capable of helping others and creating positive changes for both the employees and the organization. Therefore, they are more likely to experience a higher level of self-efficacy, enabling them to feel competent to effect change or exercise control in their environment (Rosso et al., 2010). In addition, this sense of self-efficacy enhances their sense of meaning at work (Lam et al., 2016).

This study also assumes that those who participate in OCBs are more likely to receive interpersonal cues from leaders or colleagues, which will also provide a more meaningful experience at work. For example, when managers witness an employee’s OCB, they may praise the employee and associate their OCB with organizational values that reinforce these pro-social behaviors, thereby enhancing employees’ perceptions of the meaningfulness of work. Similarly, when colleagues directly benefit from OCB, they may express sincere gratitude, further enhancing their personal sense of meaningfulness (Lam et al., 2016). The more an employee engages in pro-social behavior, the more they will perceive their job as meaningful, valuable, and worthwhile (Contreras-Pacheco et al., 2021), which, in turn, is likely to lead to improved psychological and physical health (Lease et al., 2019).

Furthermore, from the conservation of resources (COR) theory’s perspective, OCB is a positive interpersonal activity that generates positive psychological resources and may improve positive emotions (Chen et al., 2020). As mentioned earlier, OCB enables employees to realize that they can control or influence changes in the organizational environment. It also helps employees establish more meaningful interpersonal interactions to meet personal relatedness demands (Kaur and Kang, 2019). This means that positive events, such as OCB, build resources by fulfilling an individual’s needs for relatedness and competence. When a person fulfills their needs and meaningfulness, their affective well-being will increase (Kerulis, 2018).

Employees can observe, experience, and interpret more ethical behaviors when immersed in a strong ethical climate within an organization. They can learn and behave in accordance with an organization’s ethical climate, such as participating in pro-social behaviors like OCB (Aloustani et al., 2020). OCB also helps employees establish more meaningful interpersonal interactions, helping them realize that they can create positive changes for others and the organization. These positive experiences will increase the meaning of work, enrich personal resources, and improve personal affective well-being. Therefore, our third hypothesis states that OCB is a mediating variable in the relationship between ethical climate and well-being.

*H3*: OCB mediates the relationship between ethical climate and employees’ affective well-being.

### Moderation of moral identity

Moral identity is “a self-conception organized around a set of moral traits,” representing the embedding degree of morality in one’s self-awareness (Aquino and Reed, 2002). Fundamentally, an individual’s moral identity seeks answers to the question, “Am I a moral or immoral person?” (Zhu et al., 2011, p. 151). The attributes of moral identity, therefore, are individual characteristics. Previous research indicated that the impact of ethical climate on employees will differ depending on their personal characteristics (Al Halbusi et al., 2020). Thus, we will use moral identity as a moderator variable to explore whether the impact of ethical climate on employees’ attitudes and behaviors varies with the degree of an employee’s moral identity. Moral identity, which is a crucial part of the personal moral “self,” acts as an essential self-regulatory mechanism for moral behaviors (Jennings et al., 2015). In this regard, and based on the motivation of self-consistency, individuals act in ways that are consistent with how they see themselves (i.e., their identity; Chuang et al., 2016). According to social learning theory (SLT), employees with a higher moral identity are more likely to notice and act on moral cues from their environment, fostering their social learning from their surroundings. This is because those with a high moral identity will be more sensitive to relevant moral cues in the context (Hannah et al., 2011), which is important since SLT states that capturing the attention of observers is the first and most important step in observational learning. In contrast, employees with a lower moral identity possess less moral content in their self-concepts. They would therefore be less likely to activate the moral model and make ethical factors in this context less salient (Wang et al., 2019). Furthermore, moral identity increases an individual’s sensitivity to the moral factors in their environment and the degree to which they attach importance to these factors, thereby enhancing their moral evaluations (Kurpis et al., 2008). Previous research has shown that employees with strong moral identities when working within a strong ethical climate engage in less unethical behavior than employees with lower moral identities (Ge, 2018). Gerpott et al. (2019) point out that moral identity positively promotes OCB among employees. Therefore, this study believes that employees with high moral identity will identify more with the organization’s ethical climate and make more OCBs.

*H4*: Employees’ moral identities moderate the relationship between ethical climate and OCB such that this relationship is stronger for employees possessing a higher moral identity.

The P-O fit refers to the degree of congruence between employees and organizations regarding values, goals, norms, and beliefs (Chatman, 1989). Central to the P-O fit construct is the congruence between individual and organizational values. McCulloch and Turban (2007) believed that the actual fit between individual and organizational ethical values is an important predictor of employee attitudes. When an employee appears to have values consistent with their organization, they would perform well at their job and show positive work attitudes (Wu et al., 2020). P-O fit has a positive effect on employees’ job satisfaction (an important measure of job-related well-being). Merecz and Andysz (2012) found that P-O fit is positively related to employees’ health, reflected in the indices on mental and somatic health status. However, when employees perceive a meaningful inconsistency between their values and norms and those of their organization, the resulting dissonance will produce negative job performance and organizational results, producing negative emotions. Therefore, this study believes that when employees have a high level of moral identity in an organization with a strong ethical climate, they will feel more aligned with the values of their organization, improving their affective well-being in the workplace.

*H5*: Employees’ moral identities moderate the relationship between ethical climate and employees’ affective well-being, such that this relationship is stronger for employees possessing a higher moral identity.

**Figure 1:**
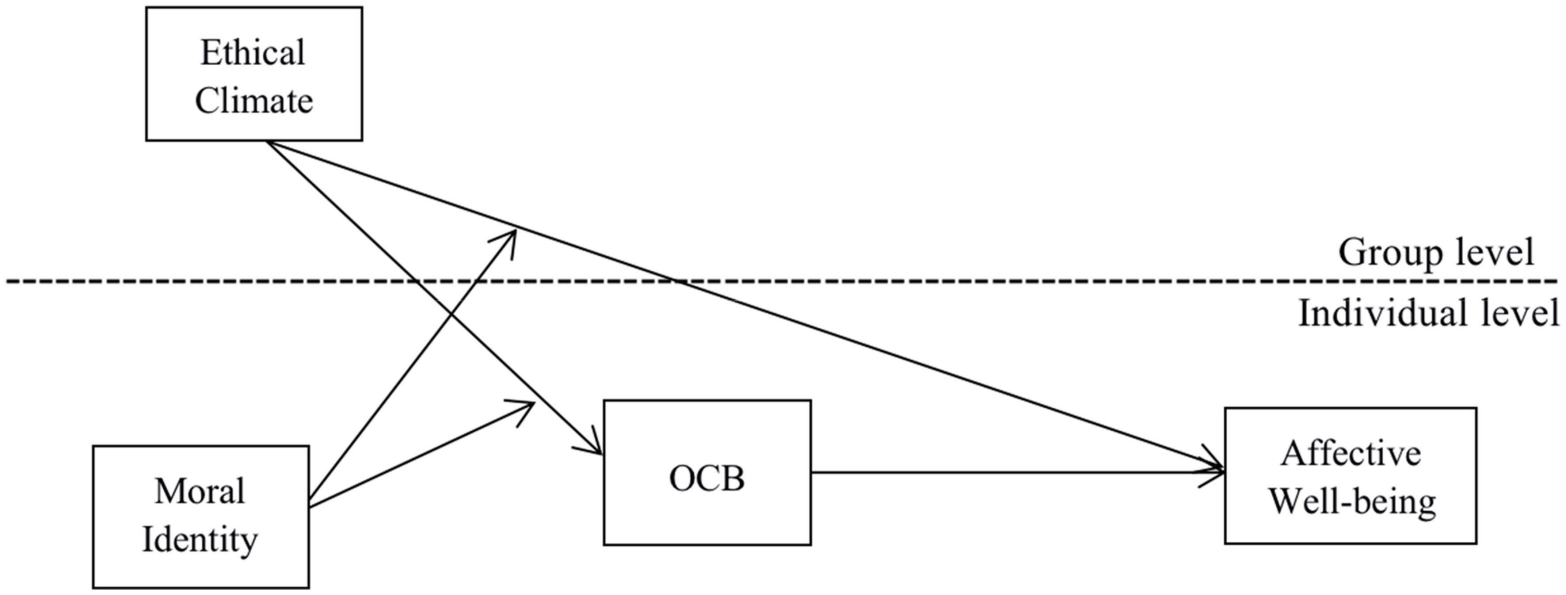
Research model.

Based on the above assumptions, we designed the research model shown below (Figure 1).

## Materials and methods

### Sample and procedure

Unlike large-scale enterprises, smaller and medium-sized enterprises tend to ignore ethical issues. According to the National Bureau of Statistics of China, two of the three provinces that have the highest numbers of small and medium-sized enterprises (SMEs) are Jiangsu Province and Zhejiang Province. The combined number of SMEs in these two provinces accounts for nearly a quarter of SMEs in China (source)^1^, and it was from SMEs in these two provinces that we collected our data.

First, we contacted each company’s human resources (HR) department. Then, with the consent of the team leader, the HR department provided the team leader’s contact information. We distributed questionnaires, explained the purpose of our research on the first page of the questionnaire, and guaranteed anonymity and confidentiality for all respondents. After the participants had read and agreed with the content of the first page, they completed the questionnaire. When the team leaders received the questionnaire, we provided them with different team numbers. We then asked them to inform their team members of their team number so that they could answer the question “Please write down your team number” in the questionnaire. In this way, we used the team number to match the data of team members and team leaders. Data were collected from two sources (team leader and team member) in two phases (four weeks apart) to minimize common method bias. Between the 11th and 18th of January 2021, team leaders were asked to rate their team’s ethical climate, and team members were invited to evaluate OCB and moral identity. We received 662 responses from 71 teams. However, after excluding incomplete and invalid questionnaires (e.g., questionnaires where the respondent selected the same option for all items), we had 526 valid questionnaires from 56 teams. Four weeks later, team members rated their affective well-being between the 15th and 22nd of February 2021, and we obtained a total of 383 valid questionnaires. After matching the last four digits of the mobile phone number in the valid questionnaire collected at the first time point with the last four digits of the mobile phone number in the valid questionnaire collected at the second time, we obtained a final total of 288 valid questionnaires from 40 teams.

We used SPSS 26.0 to analyze the descriptive statistics of the basic demographic characteristics of the valid questionnaire. Among the 40 team leaders, 77.5% (*N* = 31) were male, and 22.5% (*N* = 9) were female. Regarding the ages of the team leaders, 47.5% (*N* = 19) were aged 31–40, 42.5% (*N* = 17) were aged 41–50, and 10% (*N* = 4) were aged 51–60. Because the research object of this study focuses on millennial employees, the age of some leaders over 40 would not be an issue in this study. Regarding the team leaders’ educational level, 2.5% (*N* = 1) were high school graduates or below, 32.5% (*N* = 13) had college degrees, and 65.0% (*N* = 26) had bachelor’s degrees.

Among the 248 team members, 56.9% (*N* = 141) were male, and 43.1% (*N* = 107) were female. A total of 41.1% (*N* = 102) were aged 20–30 and 58.9% (*N* = 146) were aged 31–40. Team members’ work experience included 77.1% (*N* = 191) with 1–3 years’ experience, 21.0% (*N* = 52) with 4–6 years’ experience, and 1.9% (*N* = 5) had 7–10 years’ experience. Regarding the team members’ educational level, 1.2% (*N* = 3) were high school graduates or below, 16.5% (*N* = 41) had college degrees, and 82.3% (*N* = 204) had bachelor’s degrees.

### Measures

In this research, we adopted Schwepker (2001) seven-item ethical climate scale to measure the team’s ethical climate. A sample item was “My work team strictly enforces a code of ethics.” Participants used a five-point Likert scale (ranging from 1: *strongly disagree*, to 5: *strongly agree*) to respond to the statements. We found the scale’s reliability to be 0.904.

We also used Williams and Anderson (1991) 11-item scale to measure employees’ OCB. A sample item was “Help colleagues in work-related matters,” and was again rated on a five-point Likert scale. The scale’s reliability was 0.929.

We assessed job-related affective well-being using Warr (1990) 12-item scale with questions such as “Think about the past few weeks, how much time did you experience each of the following feelings at work:’ relaxed, enthusiastic, optimistic, cheerful, calm, contented, worried, depressed, gloomy, tense, miserable, and uneasy.” Here we asked participants to use a five-point Likert-type scale (ranging from 1 = *never*, to 5 = *always*) to respond to positive statements and another five-point Likert scale (ranging from 1 = *always*, to 5 = *never*) for negative statements. The scales’ reliability was 0.951.

Finally, we assessed moral identity using a five-item scale developed by Zhu (2008). A sample item was “I am willing to take a risk to be loyal to my moral values.” Once again, we used a five-point Likert scale (ranging from 1: *strongly disagree*, to 5: *strongly agree*), with a reliability of 0.868.

### Analysis strategy

Since employees were nested within their teams, we conducted a multi-level analysis, where we considered the team’s ethical climate as a group-level variable. Furthermore, we considered the employees’ OCB, affective well-being, and moral identity to be individual-level variables. Therefore, to confirm whether the data was suitable for multi-level analysis, we initially conducted null model testing using Mplus 8.3. An inter-class correlation coefficient (ICC) greater than 0.138 would mean that the degree of heterogeneity was high, and we could not ignore the variation of the dependent variable (Snijders and Bosker, 1999). The results of the null model test in this study showed that the ICC of OCB was 0.468, which means that, among the reasons for differences in employee OCB, 46.8% are due to differences in the ethical climate at the group-level (inter-group variation). The ICC of affective well-being was 0.446, indicating that differences in group-level ethical climate were responsible for 44.6% of differences in employees’ affective well-being (inter-group variation). Overall, these results proved the correctness and necessity of the multi-level analysis.

We then used Mplus 8.3 to perform a multi-level confirmatory factor analysis to evaluate the validity of the model construct and model fit indices. We used the following goodness of fit statistics to assess the model fitness: χ^2^/DF = 1.279 (<3), CFI = 0.980 (>0.9), TLI = 0.978 (>0.9), RMSEA = 0.034 (<0.08), SRMR within =0.041 (<0.08), and SRMR between = 0.039 (<0.08). We also used composite reliability (CR), Cronbach’s alpha, and average variance extracted (AVE) to confirm the constructs’ validity and reliability. In addition, we also conducted a multi-level path analysis to test the hypotheses, again using Mplus 8.3. Although this research utilized multi-source data to test the hypotheses, we conducted the questionnaire survey over the same period. Thus, we used Harman (1976) one-factor test in this study to check the common method variance of the data. The unrotated factor solution revealed that one factor explains 28.89% of the variance, much less than the 50% threshold, implying that common method variance was not relevant in this research.

## Data analysis and results

### Preliminary analyses

As shown in Table 1, Cronbach’s alpha values exceeded 0.70 (George and Mallery, 2003), which confirmed internal consistency for all variables. Likewise, the AVE values were above 0.50, and the CR values were above 0.70 (Hair et al., 1998). Thus, both the reliability and validity scores of the structure were acceptable.

**Table 1:** Scale reliability and validity.

Variable	Items	Factor loading	Alpha	CR	AVE
Ethical climate	My work team has a formal, written code of ethics.	0.824	0.904	0.912	0.600
	My work team strictly enforces a code of ethics.	0.815			
	My work team has policies with regard to ethical behavior.	0.671			
	My work team strictly enforces policies regarding ethical behavior.	0.896			
	I make it clear to my work team that unethical behavior will not be tolerated.	0.749			
	If an employee in my team is discovered to have engaged in unethical behavior that results primarily in personal gain (rather than corporate gain), he or she will be promptly reprimanded.	0.622			
	If an employee in my team is discovered to have engaged in unethical behavior that results in primarily corporate gain (rather than personal gain), he or she will be promptly reprimanded.	0.809			
OCB	I endeavor to keep the workplace clean and neat.	0.693	0.929	0.930	0.550
	I participate in activities organized by employee groups.	0.781			
	I make constructive suggestions.	0.678			
	I help co-workers in non-work matters.	0.820			
	I save company resources.	0.864			
	I help colleagues in work-related matters.	0.686			
	I maintain harmonious relationships and defuse conflict.	0.738			
	I prohibit behavior harmful to the organization.	0.758			
	I share useful work-related information.	0.708			
	I participate in company-organized group activities.	0.712			
	I defend the company against disasters.	0.694			
Moral identity	I view being an ethical person as an important part of who I am.	0.699	0.868	0.873	0.580
	I am committed to my moral principles.	0.787			
	I am determined to behave consistently with my moral ideals or principles.	0.865			
	I am willing to take a risk to be loyal to my moral values.	0.695			
	I am willing to place the collective interest over my own personal ego and interest.	0.748			
Affective well-being	Relaxed	0.696	0.951	0.952	0.626
	Enthusiastic	0.847			
	Cheerful	0.86			
	Calm	0.849			
	Contented	0.815			
	Optimistic	0.809			
	Worried	0.718			
	Depressed	0.768			
	Gloomy	0.777			
	Tense	0.792			
	Miserable	0.765			
	Uneasy	0.778			

Alpha, Cronbach’s alpha; AVE, average variance extracted; CR, composite reliability; OCB, organizational citizenship behavior.

Table 2 shows that all of the variables’ standard deviations were within the normal range. In addition, the research variables showed a binary correlation in the expected direction, while the square root of the AVE values displayed on the diagonal line exceeded the value of the correlations, which proved the discriminant validity (Fornell and Larcker, 1981). Therefore, the study’s data were suitable for further analysis.

**Table 2:** Correlation matrix of the study’s variables.

Variable	Mean	SD	1	2	3	4
Ethical climate	3.577	0.788	(0.775)			
Affective well-being	3.419	0.863	0.464^**^	(0.791)		
OCB	3.763	0.733	0.480^**^	0.647^**^	(0.742)	
Moral identity	3.698	0.779	0.170^**^	0.182^**^	0.154^*^	(0.762)

** *p* < 0.01;

*p < 0.05.

Square root of AVE was on the diagonal.

### Hypothesis tests

**Figure 2:**
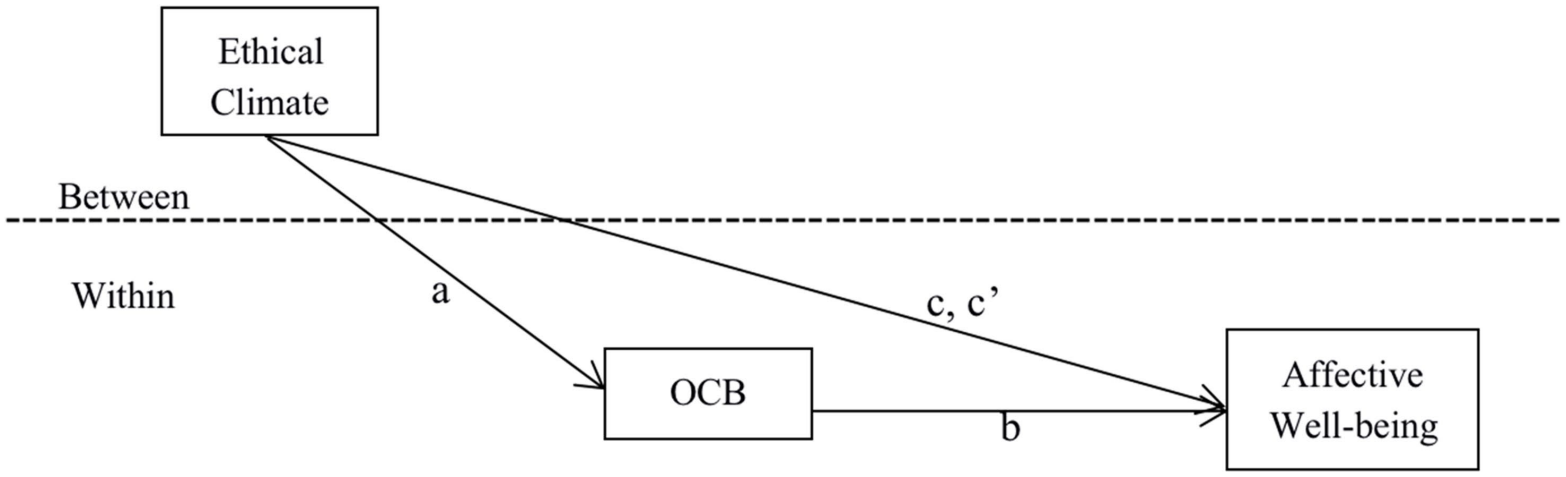
Multi-level mediation in a 2-1-1 design.

The study followed Baron and Kenny (1986) well-known methodology regarding the multi-level mediation effect by conducting four regressions to test mediating effects (Figure 2 depicts the basic causality). As shown in Table 3, the regression coefficient of ethical climate (group-level) and affective well-being (individual-level) is 0.503 (*p* < 0.001, Model 4). We also measured ethical climate and affective well-being using a five-point scale. A 1-point increase in ethical climate is associated with a 0.503-point increase in affective well-being. Therefore, this finding supports H_1_. The regression coefficient for group-level ethical climate and individual-level OCB is 0.446 (*p* < 0.001, Model 1); a 1-point increase in ethical climate is associated with a 0.446-point increase in OCB, which supports H_2_. H_3_ proposed that individual-level OCB mediates the relationship between group-level ethical climate and individual-level employees’ affective well-being. When we added the employees’ OCB (mediator) into Model 5, the positive relationship between group ethical climate and employees’ affective well-being decreased (*r* = 0.212*), while employees’ OCB was positively related to employees’ affective well-being (*r* = 0.651***). This demonstrated the partial mediation of employees’ OCB and supported H_3_.

**Table 3:** Regression analysis for hypothesis: model summary.

Level and variables	OCB				Affective well-being				
	Null model 1	Model 1	Model 2	Model 3	Null model 2	Model 4	Model 5	Model 6	Model 7
**Level 2**									
EC		0.446^***^	0.447^***^	0.446^***^		0.503^***^	0.212^*^	0.503^***^	0.503^***^
Level 1									
MI			−0.019	−0.023				−0.016	−0.018
OCB							0.651^***^		
Cross-Level									
EC × MI				−0.011					0.289 ^**^
Individual-level variance (σ2)	0.285	0.285	0.286	0.287	0.407	0.406	0.287	0.409	0.391
Group-level variance (τ)	0.258	0.135	0.135	0.135	0.337	0.183	0.129	0.183	0.185
Chi-square	257.418^***^	150.199^***^	149.844^***^	148.878^***^	243.835^***^	146.568^***^	146.285^***^	145.892^***^	152.348^***^
Deviance	468.032	451.708	453.260	458.567	553.312	537.901	452.515	539.391	534.251

*N*_level1_ = 248; *N*_level2_ = 40. Deviance is a measure of model fit; the smaller it is, the better the model fits. EC, Ethical climate; OCB, Organizational citizenship behavior; AW, Affective well-being; MI, moral identity.

* *p* < 0.05;

** *p* < 0.01;

*** *p* < 0.001.

**Figure 3:**
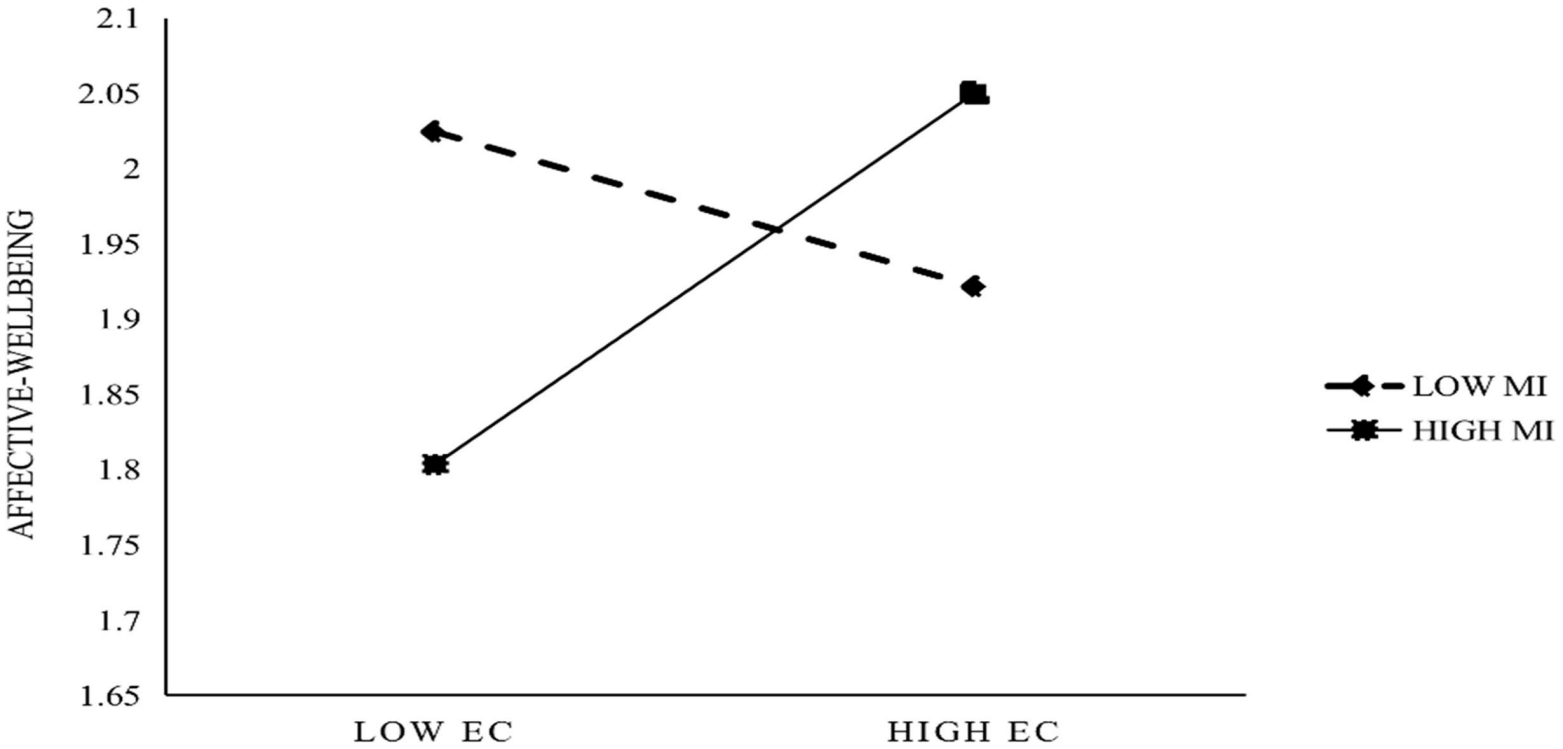
Moderation of moral identity in ethical climate and affective well-being.

To test the hypothesis of the moderating effect of moral identity, we referred to the literature regarding the use of Level 1 variables to moderate cross-level relationships (Miao et al., 2020; Saleem et al., 2020; Liang et al., 2021). We established a new interaction term (EC × MI) and entered it into the model. First, we added the moderating variable MI into Model 2 based on Model 1, but the results showed that MI had no significant effect on OCB (*r* = −0.019, *p* > 0.05). Then based on Model 3, we added an interaction term (EC × MI) to Model 4. However, the results showed that EC × MI had no significant effect on OCB, so this finding did not support H_4_. Similarly, we based Model 7 on Model 6 and added an interaction term (EC × MI). The results showed that EC × MI had a significant positive effect on affective well-being (*r* = 0.289**), supporting H_5_. As shown in Figure 3, when an organization’s ethical climate is strong, employees with high moral identity show a higher sense of affective well-being than employees with low moral identity. Interestingly, however, when an organization’s ethical climate is weak, employees with low moral identity show a greater level of affective well-being than employees with high morality.

### Robustness tests

**Figure 4:**
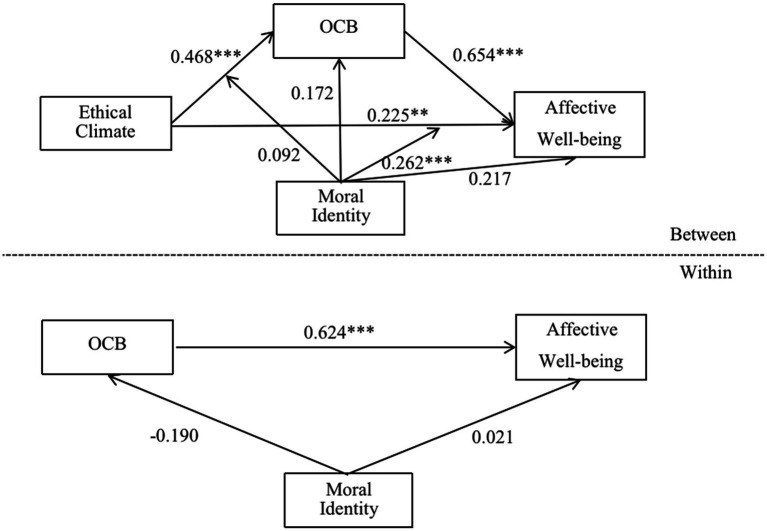
Robustness test: multi-level structural equation modeling (MSEM). ***p<0.001; **p<0.01.

This study used Mplus 8.3 for multi-level structural equation modeling to test the robustness of the results. We followed the recommendations of Preacher et al. (2010), decomposing individual-level variables (OCB and moral identity) into a within-level part and a between-level part when estimating multi-level mediation and moderation effects. For the multi-level mediation effects, we specified the fixed effects of OCB_within_ on individual affective well-being at Level 1. We also included the effects of ethical climate and OCB_between_ on individual affective well-being at Level 2. For multi-level moderation effects (Preacher et al., 2016), we included the fixed effects of moral identity_within_ on OCB and affective well-being at Level 1. At Level 2, we specified the effects of ethical climate, moral identity_between_, and one fixed interaction term (i.e., ethical climate × moral identity_between_) on OCB and affective well-being. Figure 4 shows the results of the hypotheses testing.

The direct effect of group-level ethical climate on individual-level affective well-being was 0.225, *p* < 0.01, supporting H_1_. The effect of group-level ethical climate on individual-level OCB was 0.468, *p* < 0.001, supporting H_2_. H_3_ proposed that individual-level OCB mediates the relationship between group-level ethical climate and individual-level employees’ affective well-being. The results indicated a statistically significant positive mediation effect (EC → OCB → AW) of 0.306 (0.468 × 0.654), *p* < 0.001. After adding individual-level OCB, the effect of group-level ethical climate on individual-level affective well-being was 0.225, still reaching a significant level at 0.01. This finding indicated that OCB was a partial mediation variable, and the total effect of group-level ethical climate on individual-level affective well-being was 0.531 (0.306 + 0.225). Therefore, this result supports H_3_. In addition, H_4_ and H_5,_ respectively, state that moral identity moderates the effect of ethical climate on OCB and moderates the effect of ethical climate on affective well-being. The results showed that the interaction term (ethical climate × moral identity) is not significantly related to OCB (*p* > 0.05), thereby rejecting H_4_. However, the interaction term of ethical climate and moral identity was positively and significantly related to affective well-being (0.262, *p* < 0.001). When employees’ moral identity was high, the ethical climate had a greater impact on employees’ affective well-being, meaning that this result supports H_5_.

## Discussion

This research explored the multi-level mechanism of the relationship between ethical climate (group-level) and affective well-being (individual-level) mediated by OCB at the individual-level. The model was also enriched by the moderation of moral identity (individual-level). Based on the results of empirical analysis, the main conclusions of this research are as follows. First, the group-level ethical climate has a significantly positive effect on individual-level affective well-being. Second, the group-level ethical climate has a significantly positive impact on individual-level OCB. Third, individual-level OCB partially mediates the relationship between ethical climate (group-level) and affective well-being (individual-level). Fourth, moral identity has a positive moderating effect on the relationship between ethical climate (group-level) and affective well-being (individual-level). Having a high moral identity strengthens the relationship between ethical climate and affective well-being. However, moral identity did not play a significant moderating role between ethical climate and OCB in this study. We also divided the millennial employees into two age groups (20–30 and 30–40) for additional comparison. The results showed that in the 20–30 age group, the ethical climate has a stronger positive impact on the employees’ affective well-being (0.227**) and OCB (0.694***), while the ethical climate has a slightly weaker positive impact on the affective well-being (0.155**) and OCB (0.626***) of the employees in the 30–40 age group. We can assume, therefore, that younger millennials (i.e., those in the 20–30 age group) may be more sensitive to ethical issues in the workplace. Managers should pay attention to the differences between different age groups of millennials.

### Theoretical implications

First of all, this study considered the ethical climate as an organizational variable by asking the team leader to evaluate the ethical climate of their team. The results showed that ethical climate is an effective predictor of both OCB and affective well-being. These findings are consistent with the argument suggested in previous literature that employees’ unique perceptions of their work environment and their shared common perceptions of the work environment (i.e., the organizational climate) can influence their job attitudes and behaviors (Wang and Hsieh, 2012).

Second, this study extends the literature and research on employee affective well-being in the workplace. Previous research has focused more on the impact of ethical leadership on employees’ affective well-being (Ahmad, 2019; Kaffashpoor and Sadeghian, 2020). However, ethics is a group-level phenomenon that can shape an organization’s internal relations and employee attitudes through ethical climates (Naber and Moffett, 2017). Therefore, the ethical climate is also one of the main factors shaping the organization’s internal relations and employee attitudes. The results of this study provide empirical support for the SST, responding to Newman et al. (2017) call for future research to use SST to expand our understanding of ethical climate. In particular, millennial employees who emphasize their ethical values can feel more value congruence in an organization with an ethical climate, which further boosts their affective well-being.

Third, the present findings show that OCB has a significant mediating impact on the relationship between ethical climate and affective well-being. This research conducted an in-depth study of the relationship between OCB and affective well-being, challenging the previous assumption that positive affective well-being predicts OCB. That is, OCB can also promote positive emotional experiences. This conclusion is consistent with the research results of Lam et al. (2016). Moreover, previous studies analyzed OCB from a resource consumption perspective and indicated that OCB consumes resources and thus brings negative emotions. This research indicates that OCB can also bring resources and positive emotions to people from a resource enrichment perspective. Positive psychology posits that positive and negative emotions can co-exist, and cannot be viewed as a dichotomy.

Fourth, the ethical climate can further enhance the job-related affective well-being of employees with strong moral identities. Employees who have similar values to their organization will have higher levels of affective well-being. This study verifies the moderating role of moral identity between ethical climate and affective well-being for the first time and provides empirical evidence for the P-O fit theory. Employees with a higher level of moral identity are happier in organizations with a good ethical climate. Interestingly, however, employees with low moral identity experience greater levels of affective well-being in organizations with a weaker ethical climate. When employees’ values and beliefs are in line with their organization, they can experience more positive emotions—a finding which is consistent with Al Halbusi et al. (2020) results.

Chuang and Chiu (2018) found that employees’ moral personalities positively moderated the relationship between ethical leadership and OCB. However, this study found that moral identity did not have a significant moderating effect between ethical climate and OCB. This may be a result of China’s cultural characteristics, since China has a collectivist culture whereby the organizational climate is more likely to affect the individuals (Wang et al., 2019). Therefore, even if employees have a low moral identity, they will be more affected by the organizational climate and more likely to adopt OCB in a collectivist culture. In addition, according to SLT, people will imitate and learn from their surrounding environment. Therefore, even employees with low moral identity can begin to imitate and learn the moral cues in their organization and demonstrate more pro-social behaviors if they are immersed in a strong ethical climate.

### Practical implications

First of all, HR departments should develop, clear written ethics rules outlining acceptable and unacceptable behaviors. The departments should then train or distribute information about ethics policies to employees to form common ethical values among members of the team/organization (Dinc and Aydemir, 2014). HR should also monitor ethical behaviors and investigate ethical/unethical situations since there must be a supporting reward and punishment system to reward employees for ethical behavior and sanction those who demonstrate unethical behavior. HR can increase ethical behavior by acting as an ethical role model. For millennial employees who emphasize ethics, working in a team with a strong ethical climate aligns with their ethical values and can improve their affective well-being. The COVID-19 pandemic has quickened the trend toward “working from anywhere.” In order to reduce unethical issues within a team when not directly supervised, much more needs to be done to address the ethical and compliance problems that will be pervasive in this new working environment. Therefore, it is crucial to regularly assess the ethical climate in an organization so that employees can share and discuss their perceptions (Pagliaro et al., 2018). Ethics can seem an abstract concept, but it really depends on employees’ honest and open communication with their team and with their manager.

Second, given the positive benefits of OCB, the HR department should encourage employees to demonstrate OCB through training to help new employees to socialize and integrate into the organizational environment. Through OCB, millennials can realize the value of helping others, gain more sense of work meaning, and increase their happiness levels. However, considering that OCB also has a “dark side,” the organization should give employees the discretion to engage in OCB (Kaur and Kang, 2019).

Third, the HR department can strengthen the moral identity of employees through training. It should ensure that the organization’s training program constantly instructs employees on the importance of developing ethical personalities and guides them to make ethical decisions and take appropriate actions (Chuang and Chiu, 2018). Organizations could also attempt to select employees higher in moral identity using personnel selection tools and processes (Wang et al., 2019). For example, they could conduct a situational interview to observe a candidate’s ethical decision-making when in an ethical dilemma.

## Limitations and future directions

This study first examined the impact of ethical climate and OCB on affective well-being from a holistic perspective. Based on the empirical results of this study, further research could explore the impact of various dimensions of ethical climate and OCB on affective well-being. Second, in order to better compare millennials with other generations, future research could adopt experimental designs dividing participants into two groups [e.g., a treatment group (millennials) and a control group (other generations)] to test relationships. We believe that the relationship between ethical climate and the well-being of millennials will be stronger than it is for other generations. Third, we conducted this research in China, which has a relatively homogenous culture. As mentioned earlier, China is a collectivist society, and the organizational culture is more likely to affect individuals. In more individualistically minded cultures, we believe that individuals may have a stronger sense of their own moral identity. In other words, compared with people with low moral identity, people with high moral identity will participate in more OCBs. Therefore, future research should test these possible cross-cultural differences and re-examine this research topic with millennial employees in other countries/regions to compare whether there are differences across cultures/regions; the empirical results of this study can be used as a comparative sample. In addition, the moderating effect of moral identity may also differ between different industries. For example, in industries with a high degree of teleworking, employees with a high moral identity will still demonstrate ethical behavior and “do the right thing, at the right time,” even if no one is watching. Therefore future studies can also add sector/industry characteristics to expand this research. Finally, future research can combine an organization’s ethical culture construction, a team/department’s ethical climate, and employees’ attitudes and behaviors to conduct three-level research.

## Conclusion

An important issue of human resource management is to build an organizational climate/culture that is recognized by employees within the organization. Also, with the development of positive psychology, employee affective well-being in the workplace is becoming the central topic of HRM research. Millennials have unique expectations, attitudes, and values in comparison to previous generations. They pay more attention to their well-being and their experiences of positive emotions in the workplace. Ethics are very important to millennials and are a core part of their values, meaning that they value clear ethical expectations and rules in an organization. In this study, we attempted to clarify whether millennials experience more affective well-being in organizations with a strong ethical climate that aligns with their values. A multi-level mechanism encompassing SST, social information processing theory, SLT, COR theory, and P-O fit was used to develop a model that measured the main effect of ethical climate, the mediating effect of OCB, and the moderating impact of moral identity. We found that ethical climate is a significant predictor of the affective well-being and OCB of millennial employees and that moral identity plays a partially moderated role. Previous research focused on the impact of ethical leadership, and the results of this study showed that ethical climate is also one of the main factors shaping its internal relations and the attitude of employees. Our research also indicates that OCB can also bring resources and positive emotions to people from a resource enrichment perspective. In terms of moderating the role of moral identity, it is interesting to note that employees with lower moral identity experience higher levels of affective well-being in organizations with a weak ethical climate. When employees’ values and beliefs are in line with the organization, they can experience more positive emotions, which provides empirical evidence for the P-O value fit. In summary, we encourage organizations to develop clearly written codes of ethics, regularly assess the ethical climate in their workplace, and strengthen an employee’s OCB and moral identity through constant training. Ultimately, if these measures are followed, they will significantly boost the affective well-being of millennial employees.

## Data availability statement

The raw data supporting the conclusions of this article will be available on request to the corresponding author.

## Ethics statement

Written informed consent was obtained from the individual(s) for the publication of any potentially identifiable images or data included in this article.

## Author contributions

WS and JH contributed to conceptualization, formal analysis, investigation, methodology, and writing and editing the original draft. All authors contributed to the article and approved the submitted version.

## Conflict of interest

The authors declare that the research was conducted in the absence of any commercial or financial relationships that could be construed as a potential conflict of interest.

## Publisher’s note

All claims expressed in this article are solely those of the authors and do not necessarily represent those of their affiliated organizations, or those of the publisher, the editors and the reviewers. Any product that may be evaluated in this article, or claim that may be made by its manufacturer, is not guaranteed or endorsed by the publisher.

## Footnotes

^1^ http://lwzb.stats.gov.cn/pub/lwzb/gzdt/202005/W020200603379890884764.pdf
